# PACT - Prediction of amyloid cross-interaction by threading

**DOI:** 10.1038/s41598-023-48886-9

**Published:** 2023-12-14

**Authors:** Jakub W. Wojciechowski, Witold Szczurek, Natalia Szulc, Monika Szefczyk, Malgorzata Kotulska

**Affiliations:** 1 Department of Biomedical Engineering, Faculty of Fundamental Problems of Technology, Wrocław University of Science and Technology, 50-370 Wrocław, Poland; 2 Department of Physics and Biophysics, Wrocław University of Environmental and Life Sciences, Norwida 25, 50-375 Wrocław, Poland; 3 LPCT, CNRS, Université de Lorraine, F-54000 Nancy, France; 4 Department of Bioorganic Chemistry, Faculty of Chemistry, Wrocław University of Science and Technology, 50-370 Wrocław, Poland

**Keywords:** Computational models, Protein analysis, Proteome informatics, Software, Computational biology and bioinformatics, Computational biophysics

## Abstract

Amyloid proteins are often associated with the onset of diseases, including Alzheimer’s, Parkinson’s and many others. However, there is a wide class of functional amyloids that are involved in physiological functions, e.g., formation of microbial biofilms or storage of hormones. Recent studies showed that an amyloid fibril could affect the aggregation of another protein, even from a different species. This may result in amplification or attenuation of the aggregation process. Insight into amyloid cross-interactions may be crucial for better understanding of amyloid diseases and the potential influence of microbial amyloids on human proteins. However, due to the demanding nature of the needed experiments, knowledge of such interactions is still limited. Here, we present PACT (Prediction of Amyloid Cross-interaction by Threading) - the computational method for the prediction of amyloid cross-interactions. The method is based on modeling of a heterogeneous fibril formed by two amyloidogenic peptides. The resulting structure is assessed by the structural statistical potential that approximates its plausibility and energetic stability. PACT was developed and first evaluated mostly on data collected in the AmyloGraph database of interacting amyloids and achieved high values of Area Under ROC (AUC=0.88) and F1 (0.82). Then, we applied our method to study the interactions of CsgA - a bacterial biofilm protein that was not used in our in-reference datasets, which is expressed in several bacterial species that inhabit the human intestines - with two human proteins. The study included alpha-synuclein, a human protein that is involved in Parkinson’s disease, and human islet amyloid polypeptide (hIAPP), which is involved in type 2 diabetes. In both cases, PACT predicted the appearance of cross-interactions. Importantly, the method indicated specific regions of the proteins, which were shown to play a central role in both interactions. We experimentally confirmed the novel results of the indicated CsgA fragments interacting with hIAPP based on the kinetic characteristics obtained with the ThT assay. PACT opens the possibility of high-throughput studies of amyloid interactions. Importantly, it can work with fairly long protein fragments, and as a purely physicochemical approach, it relies very little on scarce training data. The tool is available as a web server at https://pact.e-science.pl/pact/. The local version can be downloaded from https://github.com/KubaWojciechowski/PACT.

## Introduction

Pathological misfolding and aggregation of proteins is a hallmark of a number of devastating disorders, including major public health challenges, such as Alzheimer’s and Parkinson’s diseases^1,2^, type II diabetes^3,4^, and some cancers^5^. Numerous studies, both experimental and computational, have explored the mechanisms of amyloid aggregation and their roles in neurodegenerative disorders, especially the pivotal role of oligomers formed at early stages of the aggregation process^6^. These diseases not only share a similar molecular mechanism but may also cooccur in the same patient. Among others, comorbidities were observed between Alzheimer’s disease and type II diabetes^7,8^ and Alzheimer’s and Parkinson’s diseases^9^. One of the possible explanations of this phenomenon could be related to amyloid cross-interactions.

Amyloids are insoluble protein aggregates characterized by exceptional stability due to the tight packing of monomers, resulting in a characteristic pattern in X-ray diffraction experiments^10^. Typically, despite significant structural similarities shared by all amyloids, their sequences are surprisingly diverse and have little homology^11^. On the other hand, very similar sequences may also result in distinctive structures^12^. It was also shown that polymorphism of an amyloid structure may play a role in aggregation processes^13,14^.

More recent studies revealed that the presence of amyloid aggregates could affect the aggregation rate of another protein^15^. Furthermore, it was observed that interacting proteins could form heterogeneous fibrils consisting of both interacting partners. Hypothetical structural mechanisms of cross-seeding, depending on the nature of interactors, were proposed by Ivanova et al.^16^. Among others, it was shown that proteins showing sequence similarities are more likely to interact; however, many counterexamples were also found^17^. The studies highlight the importance of the structural compatibility of amyloid cores. Notably, aggregation and coaggregation can be affected by environmental or experimental conditions. In the case of conditions hampering aggregation, coaggregation may help to overcome the energy barrier needed for fibrillation, as was observed for bovine serum albumin (BSA) protein in the presence of hen egg white lysozyme (HEWL) protein^18^.

Cross-interactions were identified between numerous proteins, including those involved in type II diabetes and neurodegenerative diseases, for example, interactions between alpha-synuclein and human islet amyloid polypeptide (hIAPP)^19^. This shed new light on potentially new aspects regarding the origin of the disease comorbidity^17^. A similar mechanism was found to enhance the virulence of HIV virus by increasing its adhesion to host cells^20^. In recent years, numerous studies have also highlighted the connection between the gut microbiome composition and the onset of some diseases, including neurodegenerative Alzheimer’s and Parkinson’s diseases^21^. Despite intensive research, understanding of the molecular mechanisms underlying this connection remains elusive. A possible mechanism may include protein cross-interactions. The aggregation of bacterial amyloids could enhance the aggregation of disease-related proteins, potentially facilitating the disorder^22^. This hypothesis seems consistent with the results obtained by Chen and coworkers, who discovered increased production and aggregation of alpha-synuclein in rats exposed to bacterial strains producing biofilm-related functional amyloids^23^.

Despite the importance of amyloid coaggregation, its mechanisms are still poorly understood. This can be attributed to limited experimental data, and this shortage may also introduce a bias in available data. Thus far, interactions of a few well-described proteins, such as amyloid-beta (Abeta), islet amyloid polypeptide, or alpha-synuclein, have been very extensively studied, and they contributed to the majority of the data.

Experimental studies of amyloid aggregation, especially their interactions, are time-consuming and hampered by general difficulties in handling amyloids, which are related to their low solubility, rapid aggregation, and need for high purity^24^. The experimental methods can be based on amyloid binding of Congo Red (CR)^25^ or Thioflavin T (ThT)^26^, application of infrared spectroscopy or direct observations of fibrils with high-resolution microscopy techniques, including electron microscopy^27^ and atomic force microscopy^28^. Finally, advancements in nuclear magnetic resonance (NMR) spectroscopy have made it an important tool for studying aggregation at the molecular level^29^. However, currently, the usage of experimental methods for the identification of all amyloids in genome-wide studies would be impossible. To address this problem, several computational methods have been proposed, which are based on different approaches (reviewed in^30^ and^31^), starting from structural modeling^32^, statistical analysis of the sequence including FoldAmyloid^33^ and FishAmyloid^34^, physicochemical models such as PASTA 2.0^35^, and machine learning techniques such as APPNN^36^ and AmyloGram^37^. There are methods combining approaches, such as PATH^38^ and Cordax^39^. Finally, some methods, such as Aggrescan 3D^40^, utilize information about protein structure. It was also shown that bioinformatics techniques are quite robust and even capable of identifying some misannotated data despite being trained on them^41^. Unfortunately, none of these methods can predict amyloid cross-interactions.

Here, we present a new computational method, PACT (Prediction of Amyloid Cross-interaction by Threading), designed for the identification of potentially interacting amyloid pairs. The method is based on the molecular threading applied to the potential complex of interacting amyloids and the assessment of the stability of obtained molecular models. The method was tested on varied amyloid-related data, showing its good predictive power. Selected results of the modeling were also validated experimentally. Furthermore, to assess potential interactions between gut microbiome metabolites and amyloidogenic human proteins, we modeled interactions of bacterial functional amyloid CsgA with human alpha-synuclein, whose aggregation is a hallmark of Parkinson’s disease, and with hIAPP, which is involved in type 2 diabetes.

## Results

The presented method is based on the assumption that cross-interactions between amyloidogenic parts of proteins would result in a stable heterogeneous aggregate. Therefore, its model structure, obtained by threading into an amyloid fibril template, would be energetically more favorable than an equivalent model structure of a noninteracting pair. In PACT, we used Modeller^42^ software to thread a query sequence on the structure of amyloid fibrils formed by islet amyloid polypeptide (IAPP)^43^. To assess the obtained models, we proposed the use of the *ndope* score, which is a normalized version of DOPE (Discrete Optimized Protein Energy) statistical potential implemented in the Modeller software.

### PACT correctly identifies amyloid-prone regions

**Figure 1:**
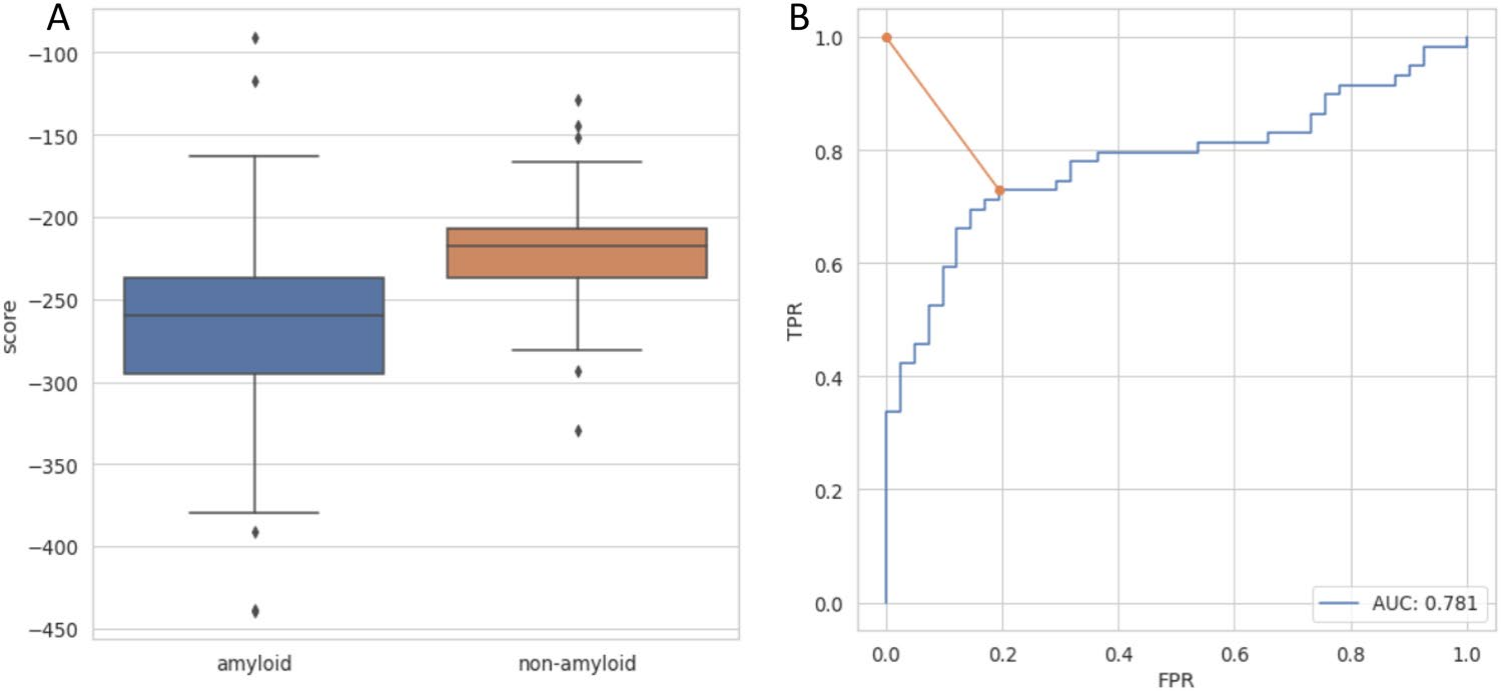
(**A**) Distribution of *ndope* score for models of amyloidogenic and nonamyloidogenic peptides. (**B**) ROC curve for amyloid vs. nonamyloid classification. The orange line represents the distance between the perfect classification point (0,1) and the chosen threshold.

In the first step, the idea presented above was tested on the homoaggregation of amyloid peptides, which can be considered a special case, and the simplified variant of interactions occurring in heteroaggregation. We compared *ndope* scores obtained for models of potential homoaggregates of amyloidogenic and nonamyloidogenic peptides, for which the sequences were obtained from the AmyLoad database^44^. Importantly, the negative dataset consisted of peptides with strong beta propensity that could be more easily misclassified for amyloid proteins by modeling methods.

The majority of models obtained for amyloidogenic peptides showed lower scores (meaning more stable structures) in comparison with nonamyloids, and their first quartiles of the scores were well separated (Fig. 1). Differences between both groups were statistically significant. Based on the Mann-Whitney U test, we were able to reject the hypothesis that the distributions of both populations were identical ($p = 2.48e-8$). Considering the energy difference, we built a threshold-based classifier. The classification threshold was chosen based on the receiver operating characteristic (ROC) curve as a point on the curve closest to the point (0,1), representing perfect classification (Fig. 1B). The optimal score value in this case equaled *ndope* = -242. If used merely for distinguishing amyloids from nonamyloids, such a classifier was able to achieve an area under the ROC curve (AUC) of 0.73 and *accuracy* of 0.77. Moreover, high values of *Sensitivity* (0.73) and *Specifictity* (0.86) were obtained. Such results are comparable with state-of-the-art amyloid predictors on the same dataset (Table S1).

We also tested whether the method is capable of recognizing amyloid propensity in functional amyloids, which poses a major problem for most predictors due to their underrepresentation in databases of amyloids. We tested the performance of the method on imperfect repeats of the CsgA protein from *Escherichia coli* and *Salmonella enterica*^45^, which were not included in our primary reference dataset (Fig. S1). Aggregation-prone regions of this protein (R1, R3, and R5) scored much lower than nonamyloidogenic regions (R2 and R4) from *Escherichia coli*. On these data, PACT achieved an accuracy of 0.9. Furthermore, the observed difference between the *ndope* score for R4 fragments from *Escherichia coli* and *Salmonella enterica* corresponds very well to the difference in their aggregation propensities observed in experiments^45^.

The results showed that the method can accurately predict aggregation-prone peptides of varying lengths. Furthermore, it potentially can be utilized to detect functional amyloids.

### PACT predicts amyloid cross-interactions

**Figure 2:**
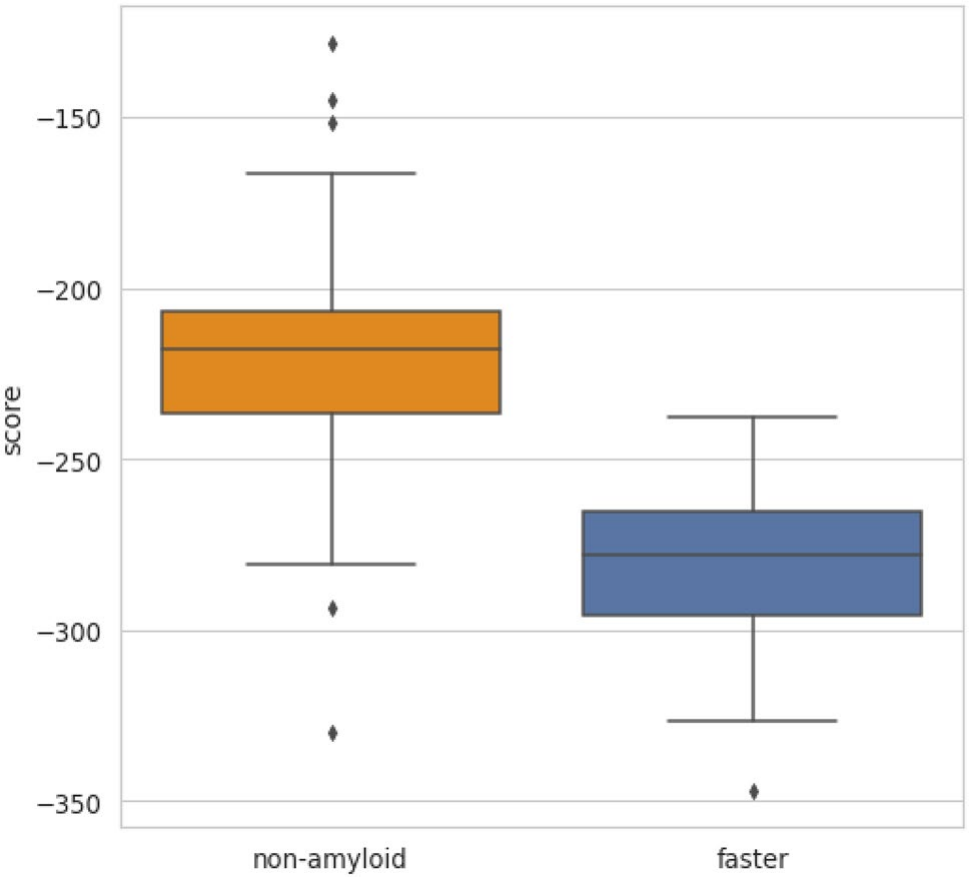
The score *ndope* for models of interacting identical nonamyloidogenic peptides (negative set) and interacting pairs resulting in increased aggregation rates (*faster* set).

We used a similar methodology to predict cross-interactions of amyloid peptides, which is the main purpose of the method. We extracted amyloid interactions from the AmyloGraph database, which contains data on interacting pairs of different amyloids^46^, and applied 119 pairs of peptides, which enhance (faster dataset) and 73, which slow down (slower dataset), the aggregation of each other. The *ndope* scores of heteroaggregates consisting of pairs of peptides whose cross-interactions resulted in faster aggregation were compared with nonamyloidogenic pairs of peptides (Fig. 2). A similar analysis was performed for pairs of peptides whose cross-interactions resulted in slower aggregation (Fig. S2). In both cases, models of heterologous aggregates resulting from cross-interactions showed lower values of *ndope* scores than nonamyloids and well-separated first quartiles of their scores (Fig. 2). Furthermore, in both cases, differences between groups were statistically significant (Mann-Whitney U test, $p = 5.54e-16$ for *faster* vs. *negative* and $p = 2.19e-15$ *slower* vs. *negative* cases. Therefore, we built the threshold-based classifier using the approach described in the previous section.

**Figure 3:**
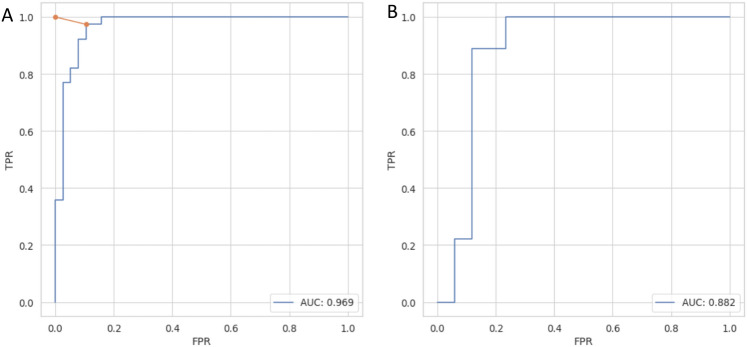
ROC curves for classification of nonaggregating and cross-interacting pairs resulting in faster aggregation on (**A**) training and (**B**) test set.

To assess the performance and choose the optimal threshold value, ROC curves were calculated for both cases: *faster* rate vs. *negative* (Fig. 3) and *slower* vs. *negative* (Fig. S3) on both training and test sets. To minimize the impact of the data choice, we performed k-fold cross-validation with k=5 on the training set and calculated several metrics describing the performance of the method (Table 1). The same metrics were then calculated on an independent test set. The same analysis was performed for the case of prediction of interactions resulting in slower aggregation (Table S2). Optimal *ndope* thresholds were very similar in both scenarios, namely, -256 and -245 for faster vs. negative and slower vs. negative, respectively. PACT performed well on both cross-validation and independent test sets. It achieved *Accuracy* values of 0.83 and 0.80 on test sets of *faster* vs. *negative* and *slower* vs. *negative* cases, respectively. In all cases, the results obtained on the test set were within the value of one standard deviation range from the mean values obtained with the cross-validation procedures. The method performance was quite similar in both *faster* vs. *negative* and *slower* vs. *negative* scenarios. However, due to the smaller dataset size, a larger standard deviation was obtained for the *slower* vs. *negative* scenario (Table 1). The results showed that the method could predict whether two peptides could cross-interact but could not distinguish between interactions enhancing and slowing fibrillation.

Finally, we tested PACT on a very limited experimental dataset of 10 nonredundant pairs of sequences from AmyloGraph (*negative set*). Here, 3 out of 10 sequences were predicted to interact, which gave a false-positive rate of 0.3, including one sequence whose score was very close to the classification threshold (see Supplementary Information, Section 6).

### PACT is robust to bias in data

A serious problem concerning the data on interacting amyloids, available in the literature and consequently our dataset, is the large overrepresentation of interactions including the Abeta peptide. This may cause overfitting of the method to Abeta. To assess the effect of the potential bias, we analyzed the scores obtained for interactions regarding different Abeta variants (Fig. S6). The observed scores for the pairs of Abeta fell within the range of values observed for the remaining pairs, and therefore, they should not have a significant effect on the performance of the method. These pairs showed a relatively narrow distribution of the *ndope* values, centered slightly below the *ndope* value of ${-275}$, which is relatively close to the identified classification threshold of ${-256}$, while the remaining interacting pairs showed even lower scores.

**Table 1:** Performance of PACT on cross-validation and independent test set for classification of non-aggregating and cross-interacting pairs resulting in faster aggregation.

	Acc [std]	Sens [std]	Spec [std]	F1 [std]	MCC [std]
Cross-validation	0.90 [0.05]	0.91 [0.03]	0.90 [0.08]	0.90 [0.04]	0.80 [0.06]
Test set	0.83	0.78	0.88	0.82	0.66

### Cross-interactions between bacterial biofilm protein CsgA and hIAPP

To further test the PACT performance and gain more insight into interactions potentially involved in amyloid diseases, the study was carried out on a functional amyloid from bacteria inhabiting human guts and human amyloidogenic protein involved in diabetes type 2. Interactions of the CsgA protein from *Escherichia coli* with the hIAPP protein were first modeled with PACT, and then the results were validated experimentally. It should be noted that CsgA protein was not included in the dataset used to develop PACT since it exceeded the maximum length of the template. It was previously shown that the whole molecule of CsgA could enhance the aggregation of hIAPP^20^. We aimed at more detailed characterization of this interaction by identifying which CsgA region is most likely to interact with hIAPP. To do so, interactions between each CsgA repeat (known for their potential amyloidogenic propensity) and hIAPP were first modeled. PACT classified positive interactions of hIAPP with its repeats R1 and R5, with scores of -257.39 for R5 and -256.52 for R1. Notably, these fragments are likely to be exposed to the environment, which also makes them good candidates for potential interactions. To test the PACT predictions, experimental validation was performed using CD spectroscopy and the ThT assay (All details regarding experimental validations and their results are presented in SI, Section 4.). Using CD, we performed preliminary tests of the propensity for aggregation of the tested peptides over time to determine the initial rate of the studied process (hours, days or weeks) (Fig S9, Table S5). The ThT assay, under varied experimental conditions, was used to evaluate the coaggregation process. The results showed stronger fluorescence and reduced lag phases for interactions with R1 and R5 fragments than those of hIAPP, R1 and R5 alone. Additionally, reduced half-lifes of hIAPP aggregation were observed in the presence of R5 (Fig. S10, Table S6) and, to a slightly weaker extent, for R1 (Fig. S11, Table S7). Importantly, the observed effects are concentration dependent, as shown in case of the aggregation rate of hIAPP upon addition of R1 fragment (Fig. S11, Table S7). This could suggest a particular role of R1 and R5 fragments in cross-seeding of hIAPP, as predicted by PACT. The results confirmed the correct prediction of PACT and showed agreement with previously published results revealing interactions of the whole protein molecules

### Mechanism of interactions between CsgA protein and alpha-synuclein

**Figure 4:**
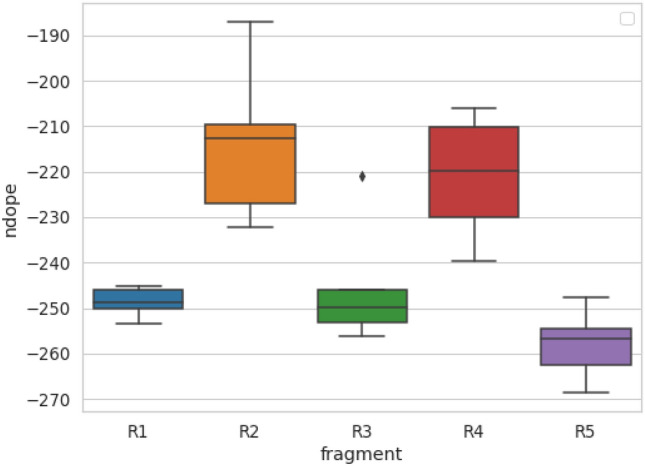
The lowest *ndope* scores indicate potential interactions of alpha-synuclein with R1-R5 repeats from a set of CsgA proteins.

Finally, to better understand a potential connection between the gut microbiome and Parkinson’s disease, we modeled interactions between bacterial functional amyloid CsgA and human alpha-synuclein, whose aggregation is a hallmark of Parkinson’s disease. The study aimed to discover more information on the interactions by specifying the unknown location of interacting regions. Here, CsgA protein originated from five different organisms found in the human microbiome: *Escherichia coli* (EC), *Hafnia alvei* (HA), *Yokenella regensburgei* (YR), *Citrobacter youngae* (CY), and C*edecea davisae* (CD). PACT was applied to predict their interactions with human alpha-synuclein, which was recently studied experimentally by Bhoite and coworkers^47^. Considering that PACT does not choose the location of the optimal fragment for automatic modeling, the sequence of alpha-synuclein was divided into overlapping fragments, each including 20 amino acids, and their interactions with R1-R5 repeats of each CsgA protein were tested. A fragment length of 20 was assumed in accordance with the length of the CsgA repeats (R1–R5). Consistent with the experimental results, all the studied CsgA variants were predicted to interact with alpha-synuclein. Among the CsgA protein fragments, R1, R3 and R5 were predicted to interact, with R5 showing the best scores (Fig. 4). These results are consistent with our current state of knowledge about CsgA, as the most aggregation-prone regions in these proteins are R1, R3 and R5. Furthermore, the R5 fragment, which showed the lowest *ndope* scores again, is typically located at the protein surface. Therefore, it can interact without the need for major conformational changes. In the alpha-synuclein part, the best scoring region was located between positions 32 and 56 (Fig. S7). This region was recently indicated as crucial for aggregation of the protein^48,49^.

### Code availability

PACT was implemented as an open-source Python module, available at GitHub repository: https://github.com/KubaWojciechowski/PACT. For users’ convenience, we prepared a docker container for the application, as well as the web server: https://pact.e-science.pl/pact/. For the prediction of cross-interaction, we recommend the use of a default score threshold of -256 and for the prediction of homoaggregation -242. The classification result denoted as “1” indicates potential interactions, and “0” indicates no interaction. Apart from the classification, the software returns generated structural models of the aggregates.

## Discussion

We proposed the first computational method, accompanied by the online tool, for predicting amyloid cross-interactions. It is based on a highly interpretable and well-established physicochemical model, which is not heavily dependent on training data. This feature is especially important since the available data contain a strong interest bias toward interactions of a few popular amyloids related to neurodegenerative diseases, for example, Abeta. However, in the case of our method, we carefully studied the effect of this overrepresentation and showed that it does not affect its performance. Furthermore, good performance on functional amyloids, which are very underrepresented in the datasets, suggests that the method is robust and can be effectively used on a wide range of sequences. In total, PACT achieved high accuracies of 0.83 and 0.80 on the independent test sets of interactions, concerning increased and decreased aggregation rates, respectively. On both sets, the method achieved high AUC values of 0.88 and 0.89 and F1 values of 0.82 and 0.77, respectively. On the other hand, since both cases were characterized by similar interaction energies, the method cannot distinguish between enhancement and inhibition of aggregation. These results suggest that these processes may be driven by similar mechanisms. This issue was addressed in a recently published work by Louros and coworkers^50^, who applied a somewhat similar approach to study the effect of point mutations on aggregation characteristics. Nevertheless, these processes may differ in more specific aspects of aggregation such as, for example, secondary nucleation.

We used PACT to predict the interactions of bacterial functional amyloid CsgA from different species with human alpha-synuclein and hIAPP. Although these interactions were not included in the training dataset, our results are in good agreement with recently published experimental data regarding these pairs of proteins. Importantly, PACT could also indicate which regions can drive the cross-interactions between both proteins, which was not previously studied. The identification of potentially interacting regions can provide important insight into the possible mechanism of the process and guide future experiments.

Apart from the identification of amyloid cross-interactions, the proposed method is also capable of reliably predicting amyloid-prone regions in proteins with accuracy comparable to that of the state-of-the-art methods. Furthermore, it overcomes their major limitations regarding the identification of functional amyloids. Unlike most of the currently available amyloid predictors, it does not rely on the scanning of a query sequence with a very short sliding window of traditionally used hexapeptides.

High-throughput identification of amyloid cross-interactions is an important step toward our understanding of its mechanisms. It can allow for a better understanding of the principles governing the process and can also be used to identify novel cases of amyloid interactions. Such capabilities can shed light on possible mechanisms responsible for the comorbidity of amyloid diseases.

## Methods

The presented PACT method is based on the assumption that cross-interactions between amyloidogenic parts of proteins would result in a stable heterogeneous aggregate. Therefore, its model structure, obtained by threading into an amyloid fibril template, would be energetically more favorable than an equivalent model structure of a noninteracting pair. A somewhat similar assumption was successfully applied in our previous work to predict the aggregation of short amyloidogenic fragments^38^. However, the current approach differs in other aspects of the method and its objectives. In PACT, we used the Modeller software to thread a query sequence on the structure of amyloid fibrils formed by the islet amyloid polypeptide (IAPP) of 37 residues^43^. To assess the obtained models, we proposed the use of the *ndope* score, which is a normalized version of the DOPE statistical potential that was implemented in the Modeller software.

### Datasets

To develop and test the method computationally, we used the following datasets:

- the set of 86 amyloidogenic (*amyloid*) and 55 nonamyloidogenic (*nonamyloid*) peptides of lengths between 14 and 45 from the AmyLoad database^44^.
- the set of 119 pairs of peptides that enhance (*faster* dataset) and 73 that slow down (*slower* dataset) the aggregation of each other. Both sets were extracted from the AmyloGraph database^46^. After the removal of redundant records, we were left with 57 and 55 pairs of peptides that enhance or slow the aggregation of each other, respectively.
- the set of 10 noninteracting pairs of amyloids, selected from AmyloGraph; the instances were filtered out from a set of 152 redundant pairs (the selection procedure described in SI, Section 6)

The first two sets (*amyloid* and *nonamyloid*) were used to test the method on cases of homoaggregation, i.e., identifying amyloid-prone peptides. The third small set was used as a negative set for additional testing of PACT.

For the prediction of cross-interactions, we used *faster*, *slower*, and nonamyloid sets. The usage of the set of nonaggregating peptides, as the primary negative set in the interaction study, was caused by the lack of a sufficient number of negative examples of noninteracting amyloid pairs. This is a common problem in studies of protein–protein interactions since negative results are rarely published, which often creates a strong bias in biological data^51^. The analysis of such a selected negative dataset, used by authors of the Tango method for identifying potential amyloid proteins^52^, revealed that it is mostly composed of peptides with strong beta propensity. The proteins from this set could be mistaken for amyloid proteins by modeling methods; therefore, they provide the best available numerous negative datasets concerning amyloidogenicity. However, for the final validation, we used the small third set of amyloids that were experimentally shown to not interact. Importantly, the CsgA protein, which was used in our final validation studies and experiments, was not included in the datasets used in the development of PACT.

The datasets used in this study are available at the GitHub repository: https://github.com/KubaWojciechowski/PACT.

### Modeling

**Figure 5:**
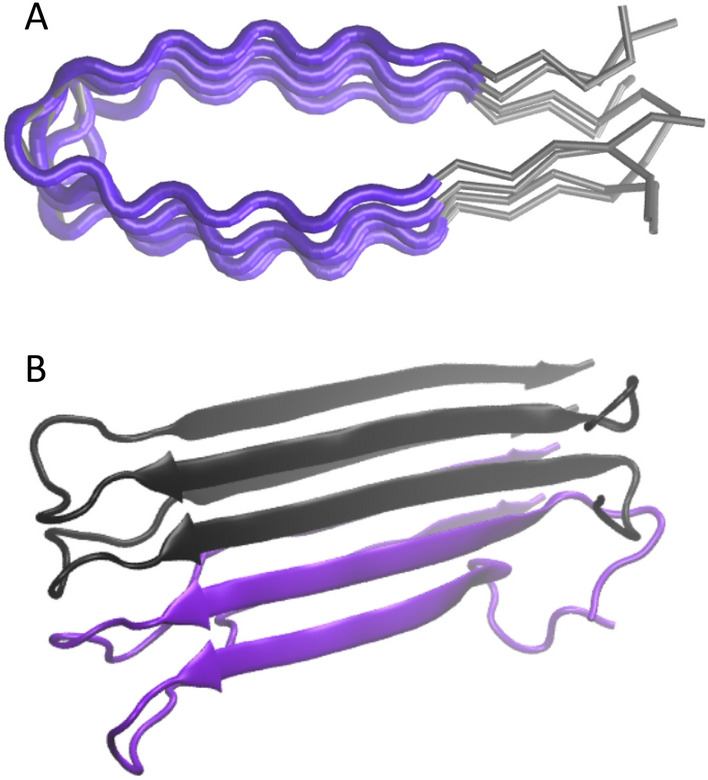
Schematic representation of the modeling procedure. (**A**) When a query sequence is shorter than the template, only a part of it is used in modeling. (**B**) The model of a heterogeneous fibril consists of two chains of each interacting peptide.

A query pair of sequences was threaded on the structure of amyloid fibrils formed by IAPP^43^. We decided to use this structure template, as it was one of the longest available structures when we started developing PACT. Since then, higher-quality templates have appeared, but their application did not improve the performance of the method (SI, Section 5). To allow the method to deal with sequences of varying lengths, sequences shorter than the template are threaded only on the main part of its structure. In such cases, a shorter sequence is aligned to the middle of the template sequence (Fig. 5A). The choice can be justified considering that most of the currently known amyloid fragments, which are longer than a few amino acids, share a similar beta-sheet turn architecture, commonly known as the beta arch. This assumption was successfully applied by Ahmed and coworkers to develop the ArchCandy method for the prediction of amyloidogenic regions^53^. PACT allows for sequences to be marginally longer than the template and, as a result, can be used to study cross-interactions between peptides of lengths between 14 and 45. Considering the presence of stochastic steps in the modeling procedure, for each of the tested pairs, 10 different models consisting of two chains of each interacting peptide (Fig. 5B) were built using the Modeller 9.24 model-multichain.py procedure with default parameters^42^. Then, the model with the lowest DOPE value was chosen for further analysis. Since the dataset consisted of fragments of varying lengths, we proposed to use the normalized DOPE score (*ndope*) defined as follows:

1 $$\begin{aligned} \textit{ndope} = \frac{DOPE}{L} \end{aligned}$$

where *L* is the average length of sequences used to build a given model. Then, *ndope* scores were compared between amyloids and nonamyloids, as well as between pairs of amyloids interacting with nonamyloids.

**Figure 6:**
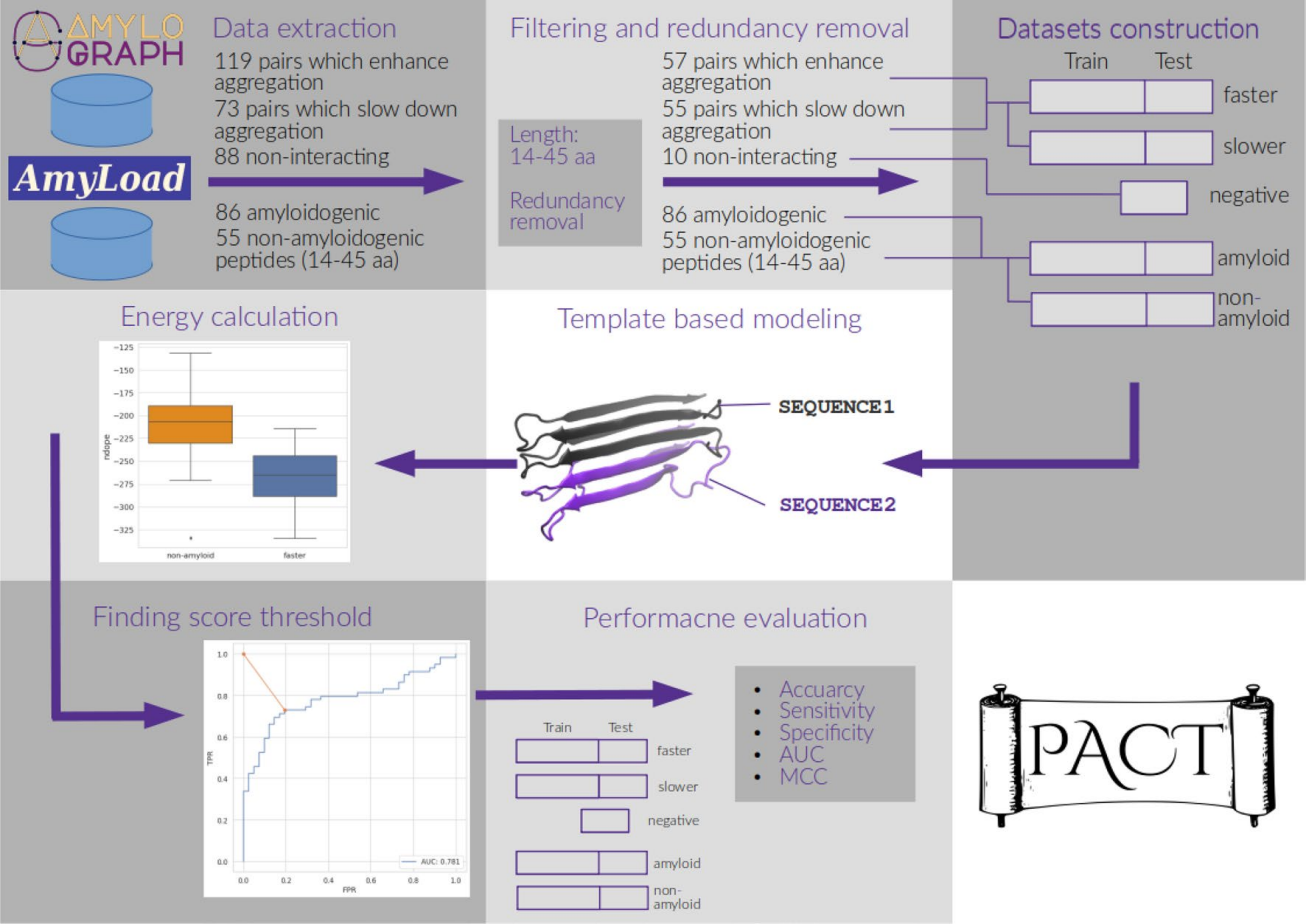
Schematic procedure of PACT.

To choose the *ndope* threshold for the classification, the ROC curve was calculated by applying different score thresholds and recording the false-positive ratio (FPR) and true positive ratio (TPR). The threshold closest to the (0,1) point (representing perfect classification) was chosen. The whole procedure is schematically summarized in Fig. 6.

We also tested a variant of the method that utilized three different structural templates (PDB: 2nnt, 2e8d); however, it did not improve the accuracy of the method but significantly increased the computational time. Therefore, this approach was finally abandoned.

### Assessment of performance and data analysis

All data analyses were performed using Python 3.8 with the Matplotlib^54^, NumPy^55^, Pandas^56^, Scikit-Learn^57^, and Seaborn^58^ packages.

To test the performance of the proposed method, a dataset was randomly split into a training set and a test set, which consisted of 30% of the data. Additionally, k-fold cross-validation (with k=5) was performed on the training data. The area under the ROC curve (AUC), *accuracy* (ACC), *sensitivity* (Sens), *specificity* (Spec) and Matthew correlation coefficient (MCC) were used to assess the performance of the method.

### Effect of amyloid-beta variants

For analysis of the effect of overrepresented Abeta pairs, we divided the *faster* dataset into two subsets: one containing only pairs where both interacting peptides were variants of Abeta (16 pairs) (*abeta*), and the set of remaining pairs (39 pairs) (*no Abeta*).

### Interactions between bacterial amyloids and hIAPP or alpha-synuclein

Modeling the interactions with between alpha-synuclein and CsgA proteins was performed using the human amylin protein hIAPP (UniProt id: P10997, fragment 34-70), the human alpha-synuclein sequence (UniProt id: P37840) and CsgA protein from five different organisms found in the human microbiome: *Escherichia coli (EC)* (UniProt id: P28307), *Hafnia alvei* (HA) (UniProt id: G9YN6), *Yokenella regensburgei* (YR) (UniProt id: A0A6H0K4L9), *Citrobacter youngae* (CY) (UniProt id: A0A549VPM7), and C*edecea davisae* (CD) (UniProt id: S3IYN9). The sequence of alpha-synuclein was divided into overlapping subsequences of lengths 20. This window length was chosen because it is similar to the length of repeated units in the CsgA protein, which is responsible for its aggregation. CsgA variants were split into nonoverlapping fragments R1-R5 corresponding to five imperfect repeats observed in their sequences. Interactions of each CsgA fragment with all alpha-synuclein fragments were studied.

### Supplementary Information

Supplementary Information.

## Data Availability

The datasets used in this study are available at the GitHub repository: https://github.com/KubaWojciechowski/PACT.
