# Supplementary material for: PACT - Prediction of amyloid cross-interaction by threading
Source: Sci Rep. 2023 Dec 14;13:22268. doi: 10.1038/s41598-023-48886-9 (PMC10721876; doi:10.1038/s41598-023-48886-9)
Supplement: Supplementary file 1 — Supplementary Information. [file 41598_2023_48886_MOESM1_ESM.docx]

Supplementary Information

PACT - Prediction of Amyloid Cross-interaction by Threading

Jakub W. Wojciechowski^1*^, Witold Szczurek^1^, Natalia Szulc^1,2,3^, Monika Szefczyk^4^, Malgorzata Kotulska^1*^

^1^ Department of Biomedical Engineering, Wroclaw University of Science and Technology Wybrzeze Wyspianskiego 27, 50-370 Wroclaw, Poland

^2^ Department of Physics and Biophysics, Wroclaw University of Environmental and Life Sciences, Norwida 25, 50-375 Wroclaw, Poland

^3^ LPCT, CNRS, Université de Lorraine, F-54000 Nancy, France

^4^ Department of Bioorganic Chemistry, Faculty of Chemistry, Wroclaw University of Science and Technology, Wybrzeze Wyspianskiego 27, 50-370 Wroclaw, Poland

* [jakub.wojciechowski@pwr.edu.pl](mailto:jakub.wojciechowski@pwr.edu.pl); [malgorzata.kotulska@pwr.edu.pl](mailto:malgorzata.kotulska@pwr.edu.pl)

1. Classification of amyloid vs non-amyloid peptides

To assess PACT performance for recognizing amyloids from non-amyloids, we calculated the same metrics for three other amyloidogenicity predictors: FoldAmyloid, AmyloGram and PACT (Table S1). PACT performance was similar to these methods, which shows that it can also be used for the prediction of amyloid-prone peptides.

Table S1. Performance of PACT on the set of aggregating and non-aggregating peptides of lengths between 14 and 45 amino acids from AmyLoad database.

| Method | Accuracy | Sensitivity | Specificity | F1 | MCC |
| --- | --- | --- | --- | --- | --- |
| PACT | 0.77 | 0.73 | 0.85 | 0.81 | 0.55 |
| PATH | 0.67 | 0.56 | 0.85 | 0.68 | 0.41 |
| AmyloGram | 0.81 | 0.83 | 0.78 | 0.86 | 0.59 |
| FoldAmyloid | 0.75 | 0.73 | 0.78 | 0.80 | 0.49 |

2. Performance on functional amyloids

Our experience shows that most amyloidogenicity predictors perform poorly on functional amyloids, which are underrepresented in available databases. To test if our method can be used on functional amyloids we tested it on R1-R5 imperfect repeats from CsgA protein from *E. coli* and *S. enterica*, which we studied previously (Szulc et al. 2021). Fig. S2 shows calculated *ndope* scores for these fragments. Aggregation-prone regions of R1, R3, and R5 scored much lower than non-amyloidogenic regions, R2 and R4, from *E. coli*. On this dataset, PACT achieved the accuracy of 0.9. Furthermore, we observed a difference in *ndope* score for R4 fragments from *E. coli* and *S. enterica, which* corresponds very well to the difference in their aggregation propensities.


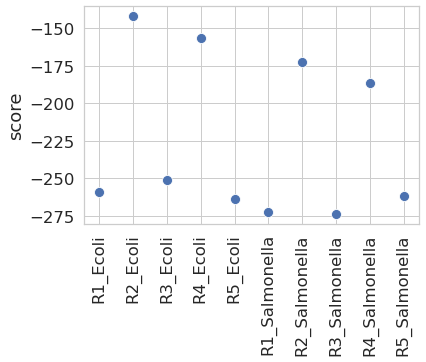


Fig. S1. Values of *ndope* scores for R1-R5 imperfect repeats of CsgA protein from *E. coli* and *S. enterica.*

3. Prediction of cross-interactions

The performance of the method on pairs of interacting amyloids was tested, using pairs whose interactions resulted in increased or decreased aggregation rate. The first case is described in more detail in the main text. Here we show the results for the case of interactions resulting in slower aggregation (Fig. S2).


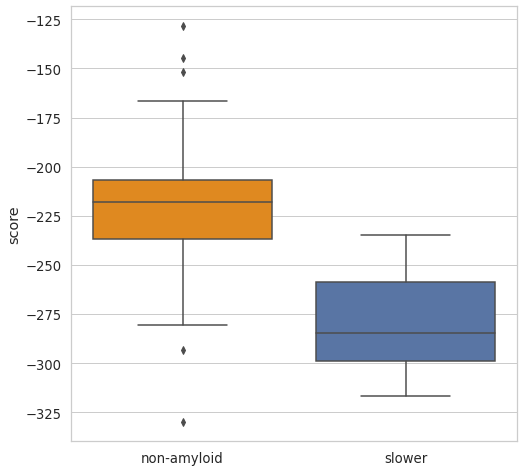


Fig. S2. Normalized DOPE score for models of non-amyloidogenic peptides and pairs of interactions resulting in decreased aggregation rates.

Similarly, as in the case of homoaggregation prediction, the threshold-based classifier was built. Here, the dataset was first split into training and test sets. Using the training set, k-fold (*k*=5) cross-validation was performed to evaluate the methodology. Then the whole training set was used to find the final threshold, and the method was tested on the independent test set. ROC curves were calculated for both training and test sets (Fig. S3). As a result, the value of -245 was selected as the optimal *ndope* threshold, and other metrics were calculated (Table S2). The results were similar to those obtained in the case of faster aggregation.


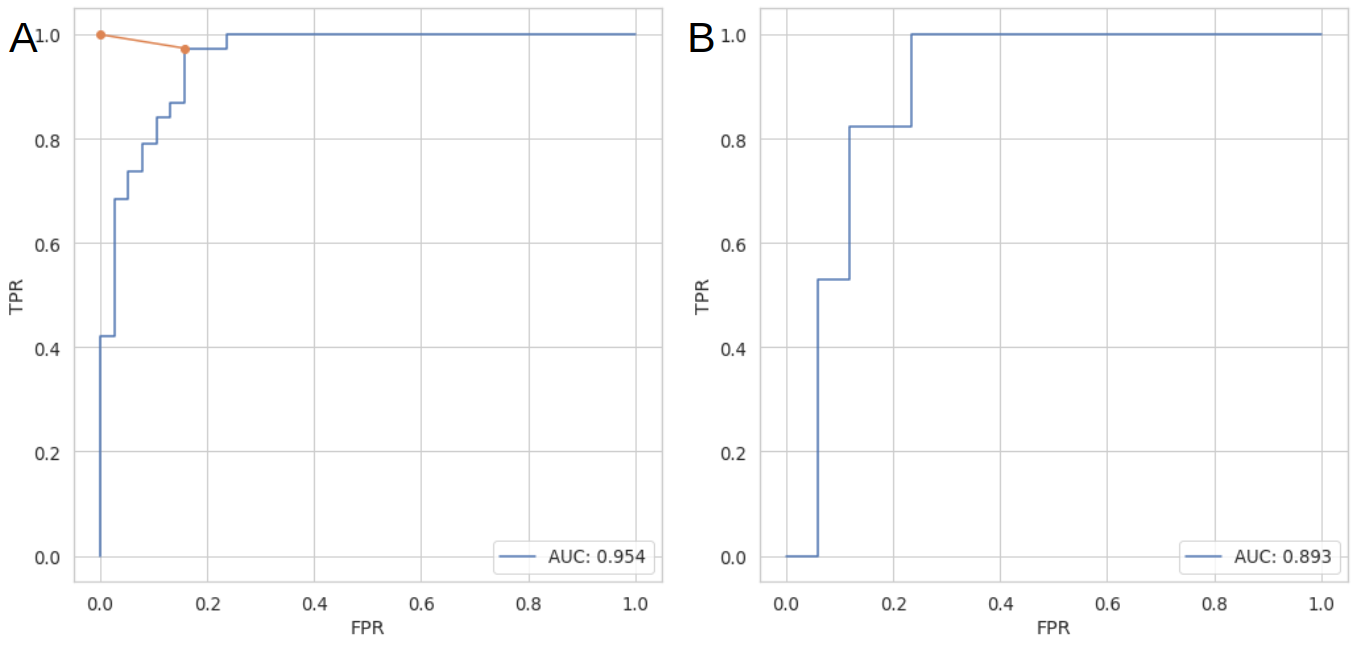


Fig. S3. ROC curves for classification of non-aggregating and cross-interacting pairs, resulting in slower aggregation, using (A) training and (B) test set.

Table S2. Performance of PACT with cross-validation and independent test set for classification of non-aggregating and cross-interacting pairs (resulting in their slower aggregation).

|  | Accuracy  [std] | Sensitivity  [std] | Specificity  [std] | F1  [std] | MCC  [std] |
| --- | --- | --- | --- | --- | --- |
| Cross-validation | 0.90  [0.07] | 0.91  [0.17] | 0.86  [0.13] | 0.89  [0.09] | 0.81  [0.12] |
| Test set | 0.79 | 0.71 | 0.88 | 0.77 | 0.59 |

We also considered the case with both *faster* and *slower* datasets simultaneously, using the same methodology. Fig. S4 shows the boxplots of *ndope* score for combined faster and slower pairs and non-amyloids. Fig. S5 shows the ROC curve for the classification. The resulting threshold is -256, and it is the same as for *faster* vs *negative*.


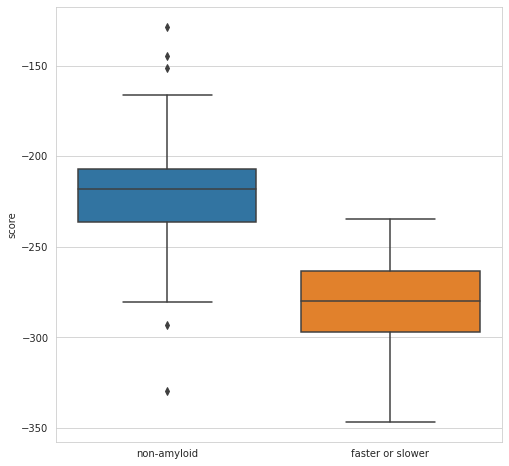


Fig. S4. Normalized DOPE score for models of non-amyloidogenic peptides and pairs of interactions resulting in increased or decreased aggregation rates.


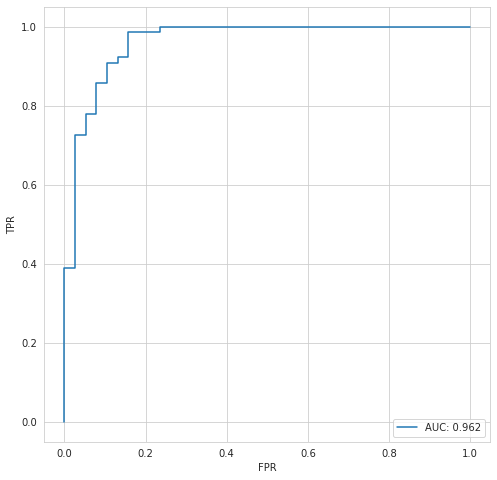


Fig. S5. ROC curves for classification, non-aggregating vs. cross-interacting pairs.

Table S3. Performance of PACT classification, non-aggregating vs. cross-interacting pairs (faster or slower aggregation), independent test set.

|  | Accuracy | Sensitivity | Specificity | F1 | MCC |
| --- | --- | --- | --- | --- | --- |
| Test set | 0.83 | 0.8 | 0.88 | 0.86 | 0.65 |

Next, we tested how the interest bias of authors of the publications influences the performance of PACT. To do so, highly overrepresented interactions of different variants of Abeta were more closely studied. The obtained scores for *A*beta pairs were within the same range as values for the remaining pairs, and therefore should not have a significant effect on the performance of the method. The pairs obtained non-divergent *ndope* values centered slightly below *ndope* value of -275, which is relatively close to the identified classification threshold for a *faster vs negative* scenario (*ndope =* -256).


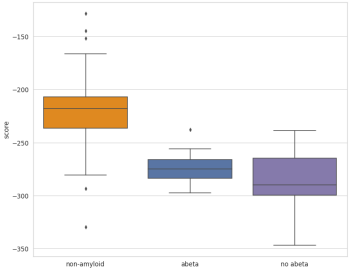


Fig. S6. Normalized DOPE score for models of non-amyloidogenic peptides and pairs of interactions resulting in increased aggregation rates, both partners belong to Abeta variants and the remaining pairs.


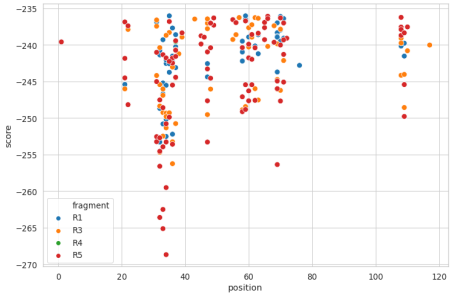

Fig. S7. Values of *ndope* score for interactions of CsgA fragments with Alpha synuclein fragments. Each dot represents the starting position of a 20-amino acid fragment of the sequence.

4. Experimental validation

The experimental validation was conducted to confirm the predictions of cross-interactions obtained with PACT. Peptides for the studies were selected based on the predicted energies. They included R1 and R5 fragments of the CsgA protein from *E. coli* species, which is a functional amyloid^1^, as well as hIAPP, an amyloid associated with type 2 diabetes^2^. The interactions between the N-terminal (R1) and C-terminal (R5) fragments of CsgA protein and hIAPP were investigated using circular dichroism (CD) and Thioflavin T (ThT) fluorescence assays. The studies were undertaken to demonstrate cross-interaction of R1 and R5 fragments with hIAPP, as predicted computationally with PACT.

Table S4. Peptides analytical data.

| **Name** | **Sequence** | **Formula** | **Calculated M/z** | **Experimental M/z** | **Analytical**  **HPLC**  **t _r_ [min]** |
| --- | --- | --- | --- | --- | --- |
| R1 | H-SELNIYQYGGGNSALALQTDARN-NH2 | C_94_H_153_N_29_O36 | [(M+2H)/2] 1228.1060 [(M+3H)/3] 819.0733 | [(M+2H)/2] 1228.1328 [(M+3H)/3] 819.0726 | 14.599 |
| R5 | H-SDLTITQHGGGNGADVGQGSDD-NH2 | C_87_H_138_N_28_O36 | [(M+2H)/2] 1994.9397 [(M+3H)/3] 997.9738 | [(M+2H)/2] 1994.2070 [(M+3H)/3] 997.5491 | 12.273 |
| hIAPP | H-KCNTATCATQRLANFLVHSSNNFGAILSSTNVGSNTY-NH2 | C_165_H_261_N_51_O_55_S2 | [(M+3H)/3] 1302.8  [(M+4H)/4] 977.3 | [(M+3H)/3] 1302.5  [(M+4H)/4] 977.3 | 10.230 |


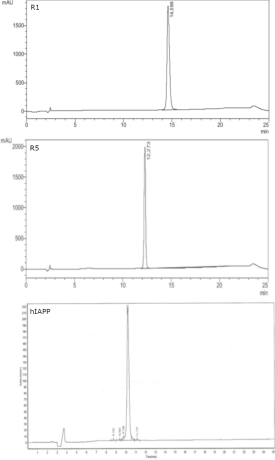


Fig. S8 Analytical HPLC chromatograms of the studied peptides.

**Materials and Methods**

**Peptide synthesis.** All commercially available reagents and solvents were purchased from Merck and used without further purification. Peptides R1 and R5 were synthesized with an automated solid-phase peptide synthesizer (Liberty Blue, CEM) using H-Rink amide ChemMatrix resin 35-100 mesh particle size (loading: 0.59 mmol/g). Fmoc deprotection was obtained using 20% piperidine in DMF for 1 min at 90 ºC. A single-coupling procedure was achieved with 0.5 M solution of *N,N′-*diisopropylcarbodiimide (DIC) and 0.5 M solution of Oxyma Pure Novabiochem® in DMF for 4 min at 90 °C. Cleavage of the peptides from the resin was accomplished with the mixture of TFA/TIS/H_2_O (95:2.5:2.5) after 3 h of shaking. The crude peptide was precipitated with ice-cold Et_2_O and centrifuged (7 000 rpm, 10 min, 4 °C). Peptides were purified using preparative HPLC (Knauer AZURA ASM 2.1L) with a C18 column (Thermo Scientific, Hypersil Gold 12 µm, 250 mm × 20 mm) with water/acetonitrile (0.05% TFA) eluent system. hIAPP was purchased from ProteoGenix, see Table S4.

**Analytical high-performance liquid chromatography (HPLC)** (Table S4, Fig. S8)**.** Analytical HPLC for R1 and R5 was performed using column ReproSil Saphir C18 100Å 5µ 4.6 × 150 mm; detection wavelength 222 nm; eluent system: A = H_2_O+0.05% TFA, B = CH_3_CN+0.05% TFA, gradient: t=0–20 min, 90%–0% A; t=20–22 min, 0% A; t=22–25 min, 0%–90% A, see Fig. S6). Analytical HPLC for hIAPP was provided by ProteoGenix and performed on PLRP-S column 100Å 4.6 × 250 mm; detection wavelength 220 nm; eluent system: A = CH_3_CN+0.1 % TFA, B = H_2_O+0.1% TFA, gradient: t=0–25 min, 10%–90% A; t=25–30 min, 100%–0% A).

**Mass spectrometry (MS).** Peptides R1 and R5 were studied by WATERS LCT Premier XE System consisting of a high resolution mass spectrometer with a time of flight (TOF) using electrospray ionization (ESI). MS analysis for hIAPP was provided by ProteoGenix.

**Circular dichroism (CD).** CD spectra were recorded on JASCO J-1500 at 20 °C between 250 and 190 nm in Phosphate Buffered Saline (PBS) with the following parameters: 0.2 nm resolution, 1.0 nm bandwidth, 20 mdeg sensitivity, 0.25 s response, 50 nm/min scanning speed, 5 scans, 0.1 cm cuvette path length. The CD spectrum of the 10 mM PBS buffer pH 7.4 was recorded and subtracted from the raw data. The peptides were dissolved in hexafluoroisopropanol (HFIP), and then mixed for 3 hours to obtain monomers. HFIP was evaporated overnight in a desiccator, then the samples were dissolved in a PBS buffer to obtain a peptide concentration of 400 µM. The CD intensity is given as mean residue ellipticity (θ [deg × cm^2^ × dmol ^-1^]). The spectra were smoothed using the Savitzky–Golay filter (polynomial order 2, widow size 19) applied in the SciPy package.

**Thioflavin T (ThT) fluorescence assay.** Kinetic measurements were carried out in a 96-well BRANDplate® on a CLARIOstar Plus, BMG LABTECH, at 20 °C, using wavelengths of 440±15 nm and 480±20 nm, for ThT excitation and emission, respectively. Additionally, the plate was shaken for 30 s at the interval of 4.92 min during 24.5 hours of measurements. The final concentrations were 50 μM of ThT and about 100 μM of each monomerized peptide’s supernatant solution after 3 minutes of 500 rpm centrifugation. Additionally, for R1 fragment the concentration of 200 μM was also used. Peptides were monomerized according to the procedure described in the CD section. The experiment was performed in duplicate. The obtained fluorescence values were normalized to the fluorescence maximum in the 0–1 range. The parameters obtained for ThT curves were fitted based on an equation of a sigmoidal function from Malmos et al. 2017 using the curve_fit function from the SciPy Python package.

**Results**CD spectra of all samples upon dissolution exhibited the minimum at approximately 200 nm (see Fig. S9 and Table S5) indicating a random coil formation. Throughout the time course of the experiment, only a slight shift towards lower wavenumbers (approximately 1-2 nm) was observed for all samples. Despite that, the observed secondary structure characteristic still resembled those of a random coil, the reduced spectral intensity and higher voltage on the photomultiplier indicated the occurrence of temporal aggregation.


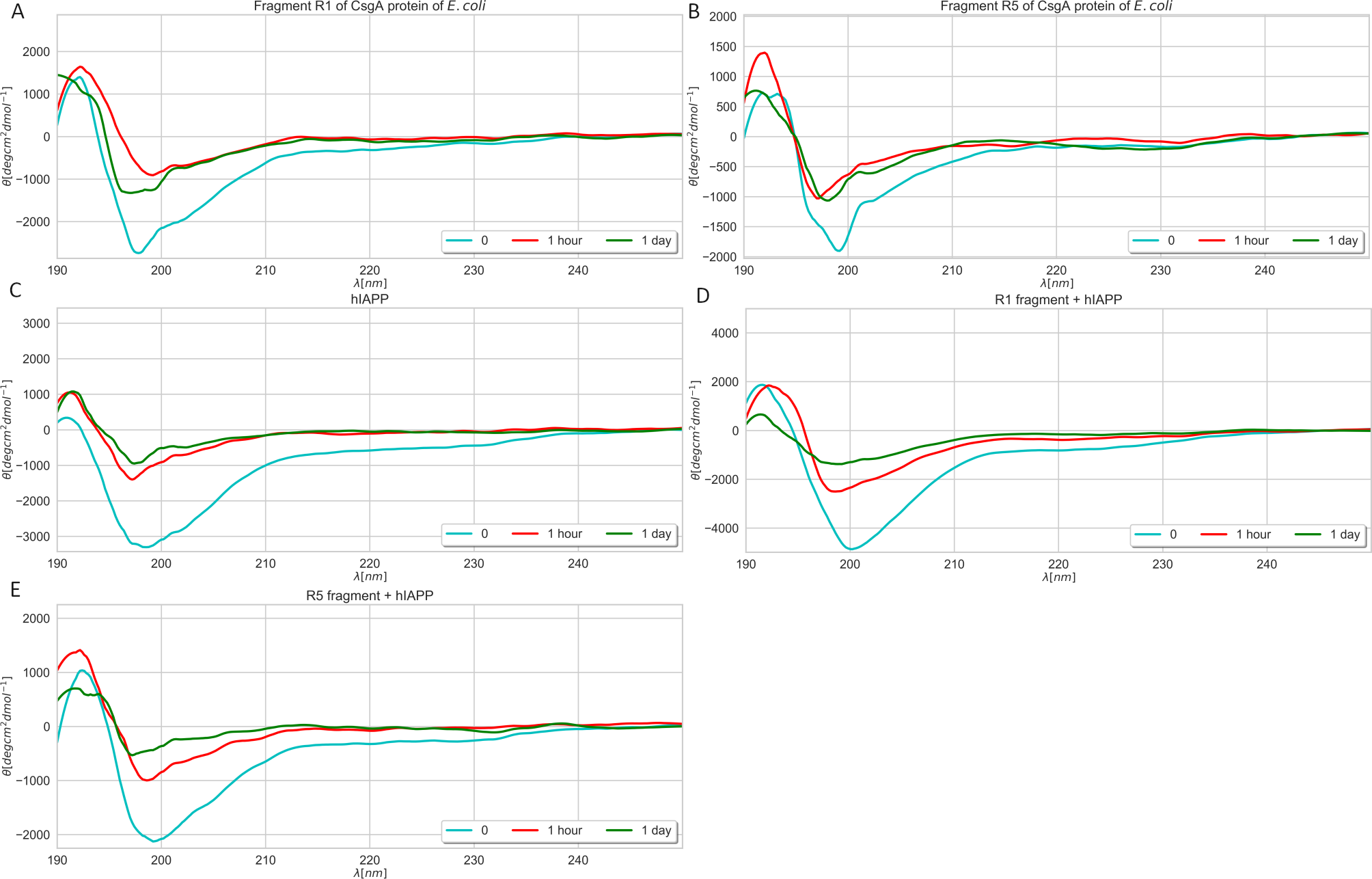


Fig. S9 Far-UV CD spectra of the studied samples: (A) fragment R1 of CsgA protein of *E. coli* species, (B) fragment R5 of CsgA protein of *E. coli* species, (C) hIAPP, (D) fragment R1 of CsgA protein of *E. coli* species + hIAPP, (E) fragment R5 of CsgA protein of *E. coli* species + hIAPP, on the day of dissolving, after one hour and after one day in the PBS buffer. C_pep_=400 µM.

Table S5 Temporal changes of the positions of CD spectra minima (in [nm]) of the samples in the PBS buffer, C_pep_=400 µM.

| **Sample** | **R1** | **R5** | **hIAPP** | **R1+hIAPP** | **R5+hIAPP** |
| --- | --- | --- | --- | --- | --- |
| **Time** |  |  |  |  |  |
| **0** | 197.8 | 199.2 | 198.6 | 200 | 199.2 |
| **1 hour** | 199.2 | 197.2 | 197.2 | 198.6 | 198.6 |
| **1 day** | 197.0 | 198.0 | 197.4 | 198.2 | 197.2 |

Comparative analysis revealed accelerated aggregation of R1+hIAPP and R5+hIAPP, compared to the individual peptides (see Fig. S10 and Table S6), which suggests cross-interactions between the peptides. The normalized fluorescence of R1+hIAPP and (especially) R5+hIAPP were higher than those of individual peptides. This was accompanied by reduction of the lag phase durations and steeper slopes. The co-aggregation effect of the C-terminal R5 fragment with hIAPP was much stronger than that of N-terminal R1 fragment, where the lag phase was reduced from 37 minutes (R5 alone) and 40.8 min (hIAPP alone) to 6 minutes (R5+hIAPP). The PACT score indicated a slightly lower energy (-257.39) for the R5+hIAPP interaction compared to R1+hIAPP (-256.52), which is consistent with the ThT results. In the concentration dependence experiments conducted on R1+hIAPP fragments, it was observed that the co-aggregation process is influenced by the concentration of the components employed (Fig. S11 & Table 7). This was corroborated by the notable reduction in the half-time of the R1+hIAPP mixture, decreasing from 49.8 to 12 minutes when the peptide concentration was doubled. The significant reduction of the t half value for the mix, compared to the values of its components at 200 µM (12 min versus 49.9 min. for R1 and 30 min. for hIAPP) also clearly showed the interactions of R1 with hIAPP.


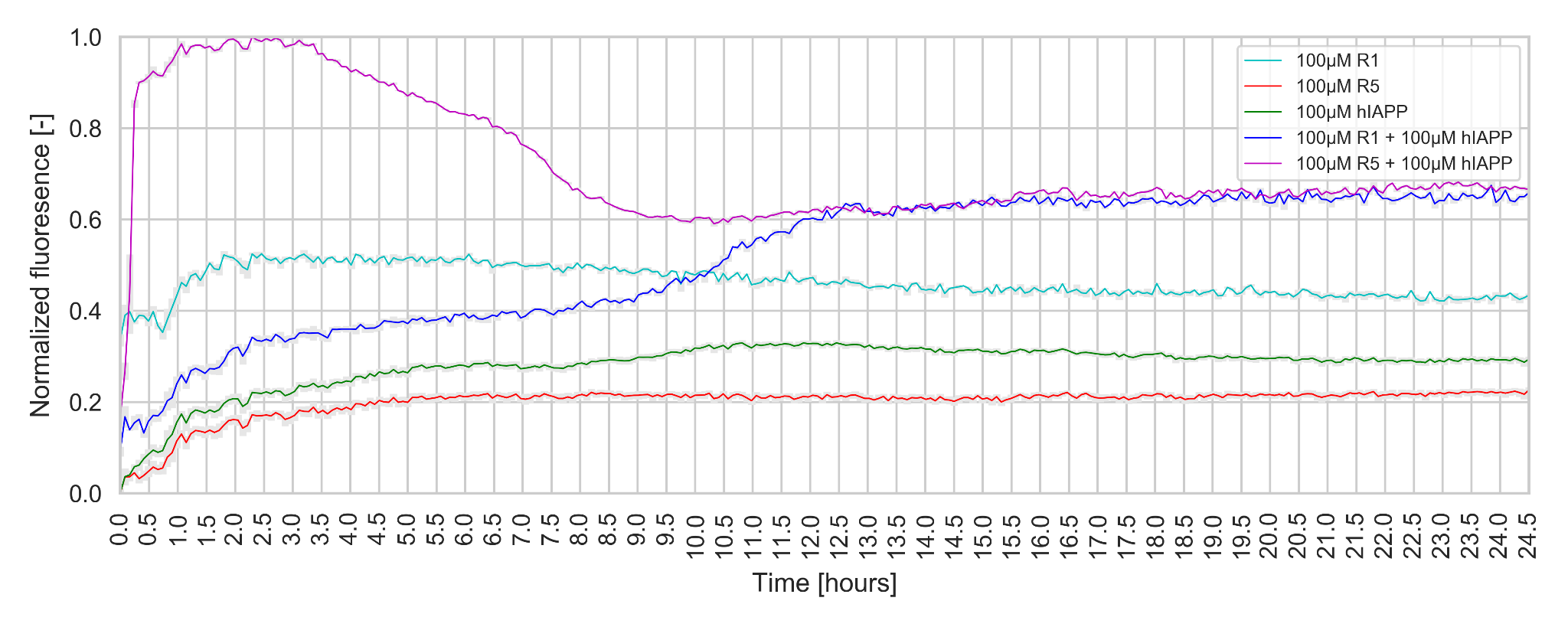


Fig. S10 ThT curves for the studied samples following the aggregation process in the PBS buffer. C_pep_=100 µM, centrifugation. Standard deviation error marked in the light gray color.


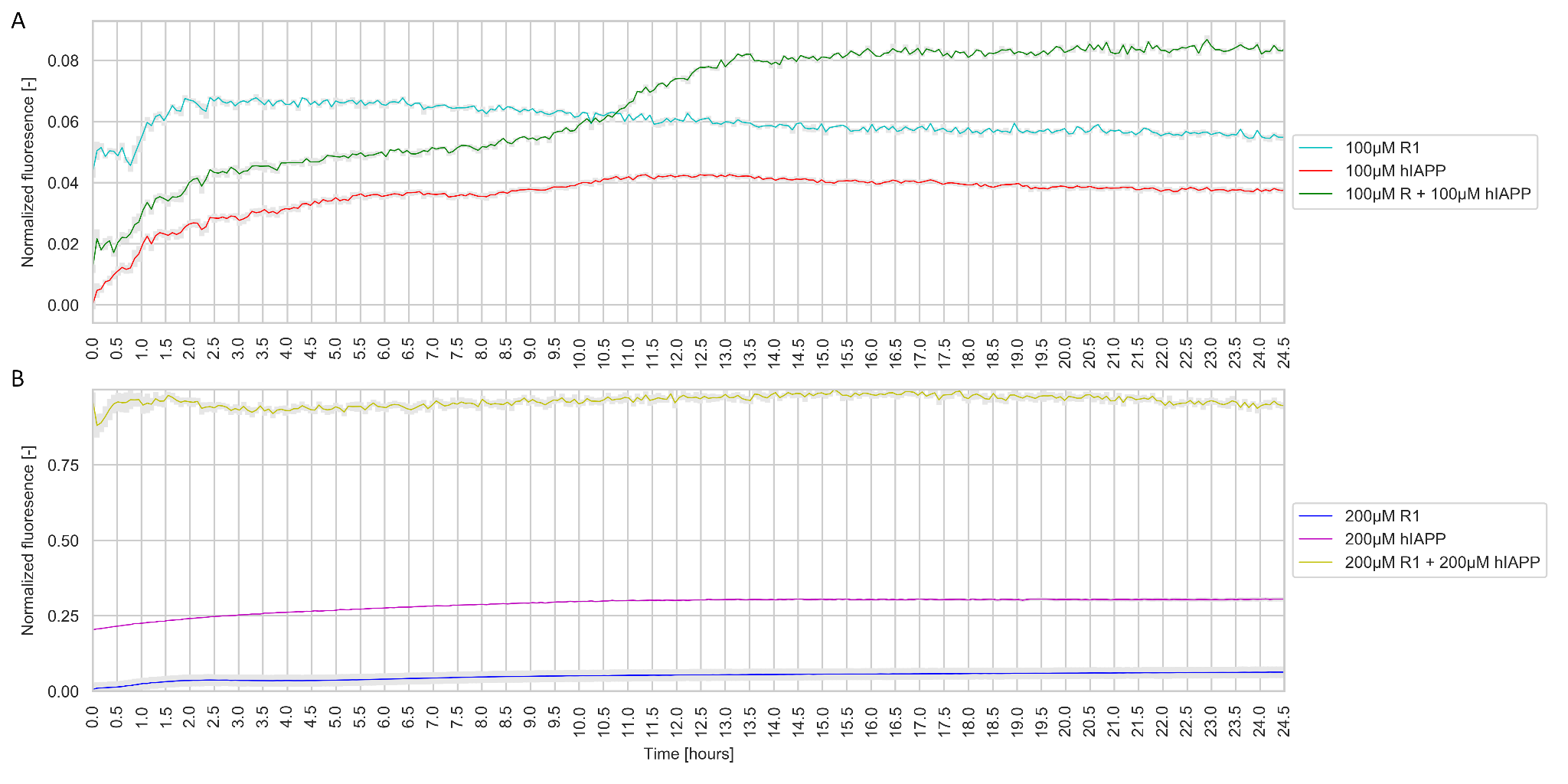


Fig. S11 ThT curves for the studied samples following the aggregation process in the PBS buffer, where (A) denotes to Cpep=100 µM, centrifugation and (B) to Cpep=200 µM, centrifugation Standard deviation error marked in the light gray color.

Table S6. Parameters obtained by fitting aggregation kinetics to studied peptides. C_pep_=100 µM, centrifugation. Here, y_i_ and y_f_ are the intercepts of the initial and final baselines with the y-axis, mi and mf are slopes of the initial and final baselines, t_half_ is the half-time in the elongation phase. The lag time is defined as t_lag_ = t_half_ − 2τ and τ is the elongation time constant (Malmos et al. 2017).

| **Parameters** | **y_i_ [-]** | **y_f_ [-]** | **m_i_ [-]** | **m_f_ [-]** | **t_half_ [minutes]** | **τ [-]** | **t_lag_  [minutes]** |
| --- | --- | --- | --- | --- | --- | --- | --- |
| **Sample** |  |  |  |  |  |  |  |
| **R1** | 0.3 | 0.4 | 0.05 | 0 | 57 | 3 | 51 |
| **R5** | 1.43E-19 | 0.22 | 0.0004 | 0 | 42 | 2.5 | 37 |
| **hIAPP** | 2.23E-14 | 0.25 | 0.14 | 0 | 46.8 | 3 | 40.8 |
| **R1+hIAPP** | 0.1 | 0.6 | 0.08 | 0 | 49.8 | 3 | 43.8 |
| **R5+hIAPP** | 0.15 | 0.6 | 1.3 | 0 | 12 | 3 | 6 |

Table S7. Parameters obtained by fitting aggregation kinetics to studied peptides. C_pep_=100 µM & C_pep_=200 µM, centrifugation. Here, y_i_ and y_f_ are the intercepts of the initial and final baselines with the y-axis, mi and mf are slopes of the initial and final baselines, t_half_ is the half-time in the elongation phase. The lag time is defined as t_lag_ = t_half_ − 2τ and τ is the elongation time constant (Malmos et al. 2017).

| **Parameters** | **y_i_ [-]** | **y_f_ [-]** | **m_i_ [-]** | **m_f_ [-]** | **t_half_ [minutes]** | **τ [-]** | **t_lag_  [minutes]** |
| --- | --- | --- | --- | --- | --- | --- | --- |
| **Sample** |  |  |  |  |  |  |  |
| **R1 (100 µM)** | 0.3 | 0.4 | 0.05 | 0 | 57 | 3 | 51 |
| **R1 (200 µM)** | 3.13E-19 | 4.47E-07 | 7.70E-04 | 0 | 49.8 | 3 | 0 |
| **hIAPP (100 µM)** | 2.23E-14 | 0.25 | 0.14 | 0 | 46.8 | 3 | 40.8 |
| **hIAPP (200 µM)** | 0.2 | 0.4 | 0.2 | 0 | 30 | 3 | 0 |
| **R1+hIAPP (100 µM)** | 0.1 | 0.6 | 0.08 | 0 | 49.8 | 3 | 43.8 |
| **R1+hIAPP (200 µM)** | 0.80 | 0.90 | 1.34E-18 | 0 | 12 | 3 | 1 |

5. Effect of modeling template

We also tested the modeling procedure using more recent templates (PDB: 6TI5 and 8BG9). The first one is a more recent NMR structure of human amyloid beta and the second one is the most recent Cryo-EM structure of the murine amyloid beta. We tested the effect of the template by modeling 45 amyloidogenic and non-amyloidogenic peptides of lengths between 14 and 45 amino acids from the AmyLoad database. The same peptides were used as a test set for the homoaggregation prediction case in the main text. We applied the same methodology as described previously, the only difference being the template. The figure below (Fig. S12) shows the correlation between model scores obtained with the previous template and the new ones. The left panel shows the correlation of scores for the new NMR structure of amyloid beta (new template) and the right one between the old template and the Cryo-EM structure (cryo template). In both cases, there are linear relationships between the scores.

**
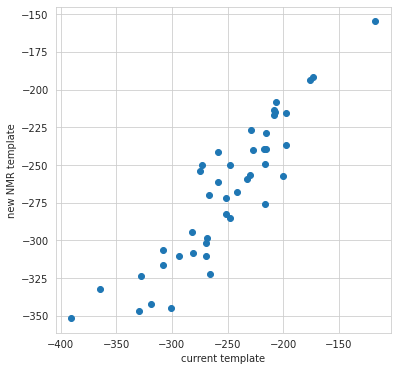

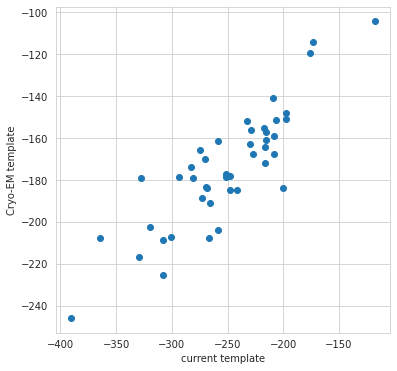
**

Fig. S12 Correlation between model scores obtained with the old template and the new ones. The left panel shows the correlation of scores for the new NMR structure of amyloid beta (new NMR template), and the right one for the Cryo-EM structure of amyloid beta (Cryo-EM template).

6. Small negative set

We analyzed non-interacting pairs included in the AmyloGraph database. First, we extracted all pairs with the “no interaction” label in the “aggregation speed” column. Initially, we obtained 152 pairs. This dataset required further processing. Some sequences were reported several times by different studies. Some of the pairs were reported to interact or non-interact, depending on experimental conditions. In some cases, one of the interaction partners was at a very low concentration. We removed these pairs and only kept instances always reported as non-interacting (88 pairs). We filtered them by length to meet the length criteria of PACT (length between 14 and 45 amino acids). After this procedure, 26 pairs remained. In the resulting set, however, there was a significant redundancy since a portion of the set consisted of mutants of IAPP. To remove the redundancy, sequences of both interactors were concatenated and clustered with CD-HIT software (Huang et al, 2010) at 80% similarity threshold. After the redundancy removal, only 10 pairs remained. We predicted their interactions using PACT (Table S8). As a result, 3 out of 10 sequences were predicted to interact, which gives a false positive rate of 0.3.

Table S8. Sequences and predictions for a set of non-interacting pairs.

| **protein1** | **protein2** |  | **score** | **classification** |
| --- | --- | --- | --- | --- |
| SSLIAGFNNSSHVLFNALRQ | QRLANFLVHSSNNFGAILSS |  | -242.82 | 0 |
| LIQSAFGNNVHLSRFNSSAL | QRLANFLVHSSNNFGAILSS |  | -234.55 | 0 |
| QRLANFLVHSSNNFGAILSS | SSLIAGFNNSSHVLFNALRQ |  | -248.17 | 0 |
| LIQSAFGNNVHLSRFNSSAL | SSLIAGFNNSSHVLFNALRQ |  | -240.03 | 0 |
| SSLIAGFNNSSHVLFNALRQ | LIQSAFGNNVHLSRFNSSAL |  | -239.87 | 0 |
| QRLANFLVHSSNNFGAILSS | LIQSAFGNNVHLSRFNSSAL |  | -252.84 | 0 |
| KTNMKHMAGAAAAGAVVGGLG | KCNTATCATQRLANFLVHSSNNFGAILSSTNVGSNTY |  | -245.39 | 0 |
| MTGLAEAIANTVQAAQQHDSVKLGTSIVDIVANGVGLLGKLFGF | MTGLAEAIANTVQAAQQHDSVKLGTSIVDIVANGVGLLGKLFGF |  | -264.08 | 1 |
| ATQRLANFLVHSSNNFGAILS | DAEFRHDSGYEVHHQKLVFFAEDVGSNKGAIIGLMVGGVV |  | -257.3 | 1 |
| KCNTATCATQRLANFLVRSSNNLGVPLPPTNVGSNTY | KCNTATCATQRLANFLVHSSNNFGAILSSTNVGSNTY |  | -299.11 | 1 |

7. All vs all amyloids

Predictions for all pairs of amyloidogenic peptides from AmyLoad were calculated to ensure that PACT does not detect the occurrence of interactions between every possible amyloid pair. The interactions were indicated for around 50% of possible pairs. Furthermore, a graph of predicted interactions was constructed, in which nodes represent sequences and edges show the predicted interactions between them. The node degree is distributed in the range of values, instead of concentrating around one value (see Fig. S13), confirming that PACT will not predict occurrence of interactions between any two amyloids.


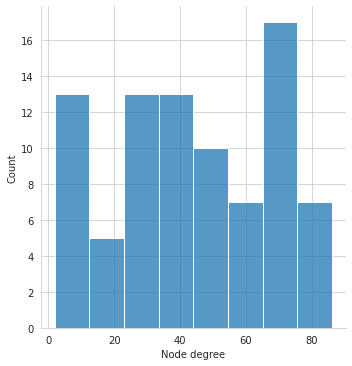


Fig. S13 Histogram of a node degree of the predicted amyloid interaction network.

**Bibliography**

Gade Malmos, K., Blancas-Mejia, L. M., Weber, B., Buchner, J., Ramirez-Alvarado, M., Naiki, H., & Otzen, D. ThT 101: a primer on the use of thioflavin T to investigate amyloid formation. *Amyloid*, **24(1)**, 1-16 (2017).

Szulc, N., Gąsior-Głogowska, M., Wojciechowski, J. W., Szefczyk, M., Żak, A. M., Burdukiewicz, M., & Kotulska, M. Variability of amyloid propensity in imperfect repeats of CsgA protein of Salmonella enterica and Escherichia coli. *International Journal of Molecular Sciences*, ***22*(10)**, 5127 (2021).

Huang, Y., Niu, B., Gao, Y., Fu, L., Li, W.. CD-HIT Suite: a web server for clustering and comparing biological sequences. Bioinformatics, (2010). 26:680
